# Caching Transient Contents in Vehicular Named Data Networking: A Performance Analysis

**DOI:** 10.3390/s20071985

**Published:** 2020-04-02

**Authors:** Marica Amadeo, Claudia Campolo, Giuseppe Ruggeri, Gianmarco Lia, Antonella Molinaro

**Affiliations:** 1 DIIES Department, University Mediterranea of Reggio Calabria, Via Graziella, Loc. Feo di Vito, 89100 Reggio Calabria, Italy; claudia.campolo@unirc.it (C.C.); giuseppe.ruggeri@unirc.it (G.R.); gianmarco.lia@unirc.it (G.L.); antonella.molinaro@unirc.it (A.M.); 2 Laboratoire des Signaux et Systémes (L2S), CentraleSupélec, Université Paris-Saclay, 91190 Gif-sur-Yvette, France

**Keywords:** Caching, Named Data Networking, Information Centric Networking, Vehicular Ad Hoc Networks

## Abstract

Named Data Networking (NDN) is a promising communication paradigm for the challenging vehicular ad hoc environment. In particular, the built-in pervasive caching capability was shown to be essential for effective data delivery in presence of short-lived and intermittent connectivity. Existing studies have however not considered the fact that multiple vehicular contents can be transient, i.e., they expire after a certain time period since they were generated, the so-called *FreshnessPeriod* in NDN. In this paper, we study the effects of caching transient contents in Vehicular NDN and present a simple yet effective freshness-driven caching decision strategy that vehicles can implement autonomously. Performance evaluation in ndnSIM shows that the *FreshnessPeriod* is a crucial parameter that deeply influences the cache hit ratio and, consequently, the data dissemination performance.

## 1. Introduction

Recent advancements in the fields of sensing, computing, communication, and networking technologies are contributing in making vehicles multi-faceted elements (equipped with cameras, sensors, radars, storage, processing, and positioning capabilities) of smart and connected cities.

Vehicular on-board units (OBUs) are able to interact among each other through vehicle-to-vehicle (V2V) communications, and with nearby road-side units (RSUs), traffic lights, pedestrians, and edge nodes through vehicle-to-infrastructure (V2I) communications. Thanks to vehicle-to-everything (V2X) connectivity, interactions with remote entities, such as cloud servers and Internet facilities, are also available. V2X connectivity would overall increase the driving and traveling experience, by enabling a rich set of applications, ranging from safety and traffic efficiency to infotainment and, more recently, cooperative and automated driving. Data exchanged by such revolutionary vehicular application will exhibit different features (e.g., size, lifetime, generation frequency, dissemination scope, popularity) and requirements (e.g., latency, throughput, reliability).

Named Data Networking (NDN) has natural advantages to greatly overcome the challenges of vehicular ad-hoc networks (VANETs), such as rapidly changing topology, harsh propagation environments and short-lived and intermittent connectivity, thanks to its name-based routing and native in-network caching capabilities [1]. Moreover, being focused on *what* content to retrieve, instead of *where* the content is located, NDN well matches vehicular applications where typically, *(i)* communicating entities are interested in retrieving content (e.g., road congestion information, weather conditions) regardless of the identity of the node(s) producing it and *(ii)* the requested contents have a spatial and/or temporal scope.

NDN implements a naïve caching strategy that lets nodes cache *all* the received contents. However, indiscriminate caching may waste network resources and reduce the cache efficiency and it is poorly suited for contents that exhibit a limited validity, which are frequently exchanged in VANET. Examples of transients contents requested by vehicular applications are, for instance, those related to parking lots availability, road congestion, maps of the surroundings [2,3]. If the content lifetime is not properly conveyed in packets and accounted for in the caching decision, stale contents risk to be propagated by affecting the behaviour of applications relying on them. Such an effect is particularly exacerbated in VANETs, due to the broadcast nature of the wireless medium that facilitates sharing of data, also of useless data, if they expired. However, literature on caching transient contents in NDN is almost unexplored in VANETs and still in its early stage for Internet of Things (IoT) contents [4,5,6] and wired networks [7,8]. To fill this gap, the following contributions are provided in this paper:

- We showcase the benefits of tracking the content lifetime in NDN packets to prevent stale information from becoming disseminated in the vehicular network.
- We propose a simple but effective NDN-compliant caching strategy that accounts for the content lifetime, not only for replacement purposes but also for the caching decision.
- We perform a comprehensive simulation campaign in ndnSIM [9], the official ns-3-based simulator of the NDN community, to study the impact of the content lifetime in the caching decision when considering two distinct vehicular scenarios, urban and highway, and varying traffic load and content popularity settings.

The remainder of this paper is organized as follows: Section 2 introduces the NDN paradigm. An overview of vehicular applications and connectivity options is provided in Section 3. Section 4 discusses the status quo on Vehicular NDN (V-NDN), with special focus on caching strategies in the literature. Section 5 motivates our study, by also providing early results, and presents the proposal. More comprehensive results are reported in Section 6. Section 7 concludes the paper with hints at future work.

## 2. NDN Basics

The NDN architecture was originally conceived for named content dissemination in the Internet [10], but today it is considered to be an enabling networking technology in different application domains, such as Wireless Ad Hoc Networks [11], IoT [12], and Edge/Fog Computing [13,14,15]. NDN is based on two packet types that carry hierarchical content names: the Interest, used by consumers for requesting contents, and the Data, used for carrying the content.

Data packets are originated by a producer/source node, which also signs them to allow per-packet authentication and integrity checks. Any node receiving Data packets can cache them to satisfy further requests. In the following, we refer to as content provider, or simply *provider*, any node in the network that acts as producer or cacher.

Each NDN node maintains three data structures: *(i) Content Store* (CS), used for caching incoming Data packets, *(ii) Pending Interest Table (PIT)*, used for recording Interests that were not yet satisfied, and *(iii) Forwarding Information Base (FIB)*, used to forward the Interests.

**Figure 1:**
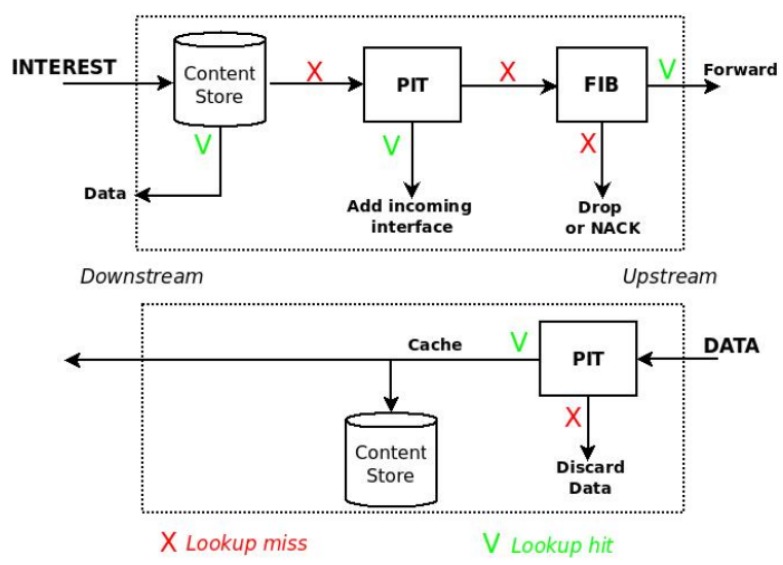
Forwarding Process in NDN.

As shown in Figure 1, at the Interest reception, each node first looks for a matching in the CS. In case of failure, it checks in the PIT for the same pending request. If a matching is found, the Interest is discarded. Otherwise, it looks in the FIB for an outgoing interface (or multiple ones) over which sending the request. Data packets follow the PIT entries back to the consumer(s); they can be cached by on-path nodes according to their storage space. The NDN reference caching implementation is Cache Everything Everywhere (CEE), where nodes cache indiscriminately every incoming Data packet. CEE is usually coupled with the Least Recently Used (LRU) replacement policy.

Compared to traditional caching systems, such as web caching or content delivery networks, NDN caching shows some distinct features: it is performed on a per-packet basis and at line speed, during the forwarding process. Therefore, cache decision policies that require complex calculations or multiple interactions between network nodes are not affordable, since they would slow down the content delivery [16].

## 3. Vehicular Applications and Connectivity Options

A plethora of heterogeneous applications will be supported in the vehicular landscape, targeting different use cases, ranging from autonomous driving to traffic efficiency and infotainment. Vehicular applications exhibit different delivery demands, e.g., in terms of latency, throughput and reliability [17] and typically exchange data with spatial and temporal relevance. For instance, road traffic information (e.g., mean speed in a given road segment) is locally relevant: data collected in one area will be requested in the same area. The time validity of such data spans a few seconds or minutes; whereas the time validity of other types of data, such as the fees of charging stations for electric vehicles and the flyers of points-of-interest in a road area, may span several hours [18].

**Figure 2:**
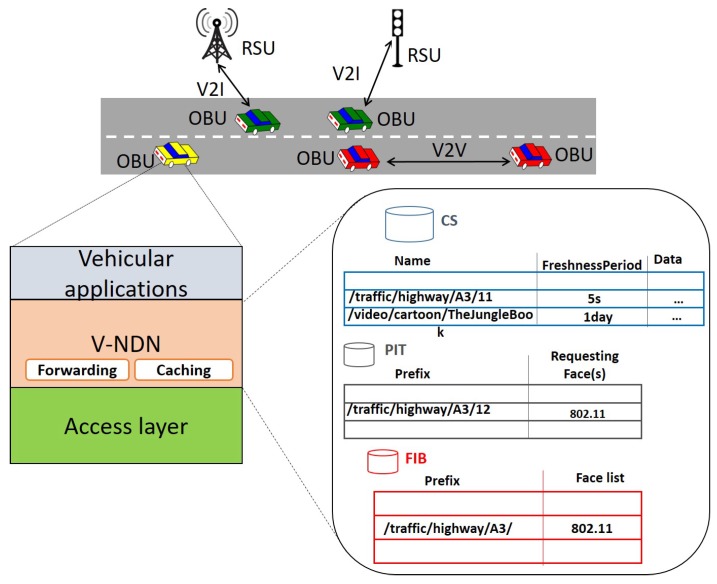
Vehicular communications and V-NDN reference architecture.

Vehicular applications rely on the exchange of data among vehicles, between vehicles and roadside infrastructure and nearby sensors, pedestrians and remote server facilities, enabled through V2X connectivity. The V2X term covers, among others, both V2V and V2I communications, as shown in Figure 2.

Although more than 20 years passed since a dedicated spectrum at 5.9 GHz was allocated to vehicular communications, the decision about the V2X radio access technology is still under debate and revolving between two mainstream technologies, i.e., IEEE 802.11 and Cellular-V2X (C-V2X).

IEEE 802.11 initially captured the interest of the research community, due to operation simplicity and native support for V2V communications in an ad hoc manner. The IEEE 802.11p amendment, now superseded and part of the IEEE 802.11 standard [19], was conceived as an enhancement of the IEEE 802.11a specification, with physical and medium access control (MAC) layers’ settings and procedures properly adjusted to support outdoor communications under high speed mobility. At the MAC layer, 802.11p relies on the Carrier Sense Multiple Access with Collision Avoidance (CSMA/CA) protocol. A node wishing to transmit senses the medium to detect if it is busy. If it is the case, a mechanism based on random backoff is performed to reduce the probability of collisions, which, however, cannot be prevented. More recently, the interest for 802.11-based V2X connectivity revamped thanks to the creation of a new IEEE task group, now preparing the IEEE 802.11bd amendment. The group aims to investigate evolved physical-layer technologies that enhance the 11p coverage and throughput [20].

## 4. V-NDN: Design Concepts and Caching Strategies

### 4.1. V-NDN

Originally designed in [21], V-NDN extends the NDN model to accommodate the distinctive and challenging features of VANETs, namely ad hoc intermittent connectivity and mobility, to fit the spatio-temporal validity of contents. A reference V-NDN architecture is shown in Figure 2. As with the vanilla NDN implementation, each V-NDN node maintains CS, PIT and FIB tables but, to take full advantage from the broadcast nature of the channel and maximize the possibility of content sharing, major modifications are introduced in the forwarding and caching process.

**Forwarding.** Due to the high dynamicity of vehicular topologies, V-NDN does not implement a proactive routing protocol to build the FIBs. Instead, it assumes that Interest and Data packets are always broadcasted over the wireless medium, and it designs a reactive distance-based forwarding scheme that limits packet collision and redundancy and speeds up the data retrieval. More specifically, when sending an Interest, each node *S* includes its Global Positioning System (GPS) coordinates. Each receiving node *R* calculates its distance from *S* and sets a random *Defer Time* that is inversely proportional to such distance. The smaller the distance the larger the time a node waits before transmitting; therefore, the farthest node from the sender has higher transmission priority. This speeds up the Interest dissemination. If, during the *Defer Time*, *R* overhears the same packet broadcasted by another node in the same area, then it can cancel its own transmission. Re-broadcasted Interests act also as an implicit acknowledgment for the sender *S*. In case no rebroadcasting is overheard, then *S* will retransmit the packet up to 7 times before giving up. To avoid the unrestrained dissemination of Interests, a maximum hop count limit is set to 5 [21].

**Caching.** Unlike other wireless terminals, such as smartphones or sensors, vehicles do not have strict energy or memory constraints. Therefore, V-NDN nodes can, in principle, cache all Data overheard over the wireless channel, even if they do not have a matching PIT entry. We call this strategy Cache Everything in the Air (CEA). By implementing CEA, vehicles can serve as data mules between disconnected areas and enable opportunistic delivery services. In practice, however, this strategy may lead to inefficient performance due to the high cache redundancy (which is even higher than CEE) and it is not convenient in areas with a high density of vehicles [22]. This is why other caching strategies were proposed in the literature, as discussed in the following section.

### 4.2. Cooperative vs. Autonomous Caching

In wired networks, cooperative schemes usually lead to better performance than autonomous ones, in terms of low cache redundancy and reduced retrieval latency, at the expense of a potentially high signalling overhead [23]. Things however change in vehicular environments where, due to node mobility and unstable connectivity, it is difficult to exchange consistent information about the status of the network and the CSs of vehicles and take decisions at line speed. Traditional approaches for ad hoc networks (outside the NDN context) such as the one in [24], which takes decisions based on information density estimated during an inference phase, seem not affordable in NDN, since they would introduce a slowdown in the forwarding fabric [16,25].

To cope with the dynamicity of vehicular topologies, some studies considered mobility-aware caching strategies that can be applied in the presence of a full or partial RSU infrastructure. In [26], a proactive caching policy is proposed that takes into account the content popularity and the vehicle mobility prediction. The latter information is used to prefetch the contents at the RSUs the vehicles will be connected to during their journey, thus their requests will be satisfied with lower latency. However, the strategy requires the collection of prior mobility data over which applying offline a mobility prediction algorithm. In [27], a scheme called Cooperative Caching based on Mobility Prediction (CCMP) is designed where urban areas are divided into hot regions based on users’ mobility patterns and a prediction model is applied to compute the probabilities that vehicles re-visit the hot regions. Nodes with higher chances of staying in hot regions and for longer times are chosen to cache contents. In [28], mobility prediction is used to create clusters of vehicles. The most suitable vehicles (e.g., the ones with the best channel quality) are selected as cluster heads and act as cachers: they receive contents from the closest RSUs and cache them to serve requests from other vehicles. A shortcoming of such approach is the lack of fairness, since only few nodes handle caching operations.

When the mobility prediction is not available, implementing cooperative caching schemes may be disadvantageous. For instance, the work in [22] shows that a notable caching scheme with implicit coordination, Leave Copy Down (LCD) [29], has results comparable to a Never Cache policy, in which vehicles do not cache packets. In the rest of the paper, we focus on a general scenario where vehicles mobility patterns are a priori unknown and communications are mainly based on short-lived V2V interactions. Autonomous caching schemes better suit this type of situation: they are lightweight solutions that do not require additional knowledge of the network topology and do not incur in further signalling.

### 4.3. Autonomous Schemes for Non-Transient Contents

CEE and CEA are the simplest autonomous caching schemes available in the literature. To limit their intrinsic cache redundancy effects while maintaining a simple decision criterion, random schemes with a static caching probability were devised [30], where nodes cache Data packets with a pre-defined probability *p*, with 0≤p≤1. If p=0 the node never caches packets, while if p=1 the scheme behaves like CEE. The most common value used in NDN implementations is p=0.5, which limits the cache redundancy without underusing the available storage space [31]. The caching probability can be also computed dynamically at each node based on the perceived information about the network status and the content demands in the neighbourhood. In this context, decision strategies largely vary depending on the communication type, namely V2I or V2V.

In [32], an autonomous probabilistic caching scheme, for RSUs only, is deployed, with the targets of minimizing the average number of hops to retrieve the requested content and maximizing the cache hit ratio. The caching probability is computed according to the content popularity evaluated adaptively to the distinct request patterns. However, the strategy is deployed in an infrastructured network of RSUs that retrieve contents from a remote server and it does not consider caching at vehicles. Conversely, in [33], the focus is on V2V communications and the caching probability is computed by vehicles according to three parameters: *(i)* the content popularity, inferred from the received Interest packets, *(ii)* the vehicle’s topological centrality, and *(iii)* the relative movement of the receiver and the sender. Performance evaluation shows that the strategy outperforms CEE and probabilistic caching with p=0.5. In [34], a cache probability utility function is defined that takes into account the content popularity and two new defined parameters, the *moving similarity* and the *content similarity*. The moving similarity indicates if a content is requested by vehicles moving on the same route of the potential cacher. The higher the moving similarity the longer the connectivity between vehicles, and therefore the higher the caching probability. Vice versa, the content similarity indicates if drivers/passengers request similar contents. The higher the content similarity the higher the probability that a vehicle will cache a received Data packet.

## 5. Caching Transient Contents in V-NDN

### 5.1. Contributions of This Paper

The above-mentioned caching schemes do not consider that many contents exchanged in a vehicular environment have a limited time validity, which can vary from a few seconds to a couple of minutes. Intelligent driving assistance systems, parking lots availability, high-definition maps are just a few examples of services that rely on contents that may change with time due to the variation of the driving environment [2,3]. They entail fresh content retrieval and also short latency. Such a transient feature may largely influence the performance of a caching system and cannot be ignored in the caching decision. Therefore, in the following sections, we aim at exploring the impact of transient contents in the caching systems of V-NDN nodes. In particular:

- We detail how the vanilla NDN forwarding fabric deals with transient data, by emphasizing the potential weaknesses, and report the few related literature studies in the field.
- We perform a first basic simulation campaign to quantify the effects of caching transient contents in V-NDN by studying crucial metrics such as the cache hit ratio and the cache inconsistency, which is due to the wrong awareness about the content lifetime supported by the vanilla NDN implementation.
- We design a new autonomous strategy, named Freshness-Driven Caching (FDC), which addresses the cache inconsistency issues and takes caching decisions based on the content lifetime. The rationale behind our proposal is pretty intuitive: caching contents that are ready to expire (possibly at the expenses of contents with a larger lifetime) does not efficiently use the storage space. Indeed, regardless of the request pattern, short-lasting contents will be quickly removed from the CS. Therefore, FDC aims at caching long-lasting contents with a higher probability.
- Performance of FDC is evaluated in two different mobility scenarios, urban and highway, and compared against two NDN benchmark schemes, CEE and random caching.

### 5.2. Cache Inconsistency in Existing Solutions

In mobile wireless and broadcast environments such as VANETs, vehicles act like data mule that collect packets and move them across distances thus offering a valuable dissemination service. Transient data, however, require an ad hoc caching strategy that is aware of their lifetimes. Storing stale data can lead to cache inconsistency, i.e., distinct cachers can have inconsistent copies of the same content that result in multiple adverse effects in the real life. For instance, if a vehicle looks for an empty space by transmitting an Interest in its neighborhood, it may uselessly reach a wrong (busy) space, by wasting both fuel and time, if stale data are received as a reply.

In vanilla NDN, the transient feature of a content is expressed in term of a *FreshnessPeriod* (in the following shortened as FP), a field in the Data packet header indicating the lifetime of the content. The parameter is application-specific and it is set by the original producer. If the lifetime is not expired, the content can be considered still *fresh*. Otherwise, the original source may have produced new content. Tracking the freshness is, therefore, crucial in presence of transient data not to incur in cache inconsistency effects. A policy that honors content freshness (we refer to as “FP-Aware” policy) is implemented in vanilla NDN and applied in conjunction with a standard replacement policy such as LRU. Basically, when caching a received Data packet, the NDN node sets a timer equal to the FP value; when this latter expires, the content is removed.

Literature on caching transient data in NDN is still at its infancy and almost unexplored in vehicular environments. Some proposals were devised in IoT sensor networks, with the main target of reducing the data retrieval latency and limit the energy consumption [4,5,6]. Other works considered caching transient contents in wired networks segments [7,8].

With focus on V-NDN, a Multi-Metric Cache Replacement ($M^{2}CRP$) scheme is presented in [25]. There, content popularity, freshness, and distance between the content producer and the cacher are used to select the packet that must be evicted from the CS. $M^{2}CRP$ is coupled with the CEE policy: when a new Data packet is received, it is always cached and, if the CS is full, then an existing cached item must be replaced. Popularity, freshness and distance metrics are used to compute a score for every cached item; the one with the minimum score is selected as the candidate for eviction. A similar replacement strategy, but implemented in RSUs only, is deployed in [35]. Works [25,35], however, do not consider the effect of freshness in the caching decision process. By using CEE, all the contents are indiscriminately cached regardless of their freshness period. On the one hand, CEE does not create content diversity within the vehicles’ neighbourhood: the CSs of vehicles in the same area are filled with the same information, thus resulting in an inefficient use of the distributed storage space. On the other hand, caching contents with a long lifetime could be more convenient than caching contents with a shorter lifetime, since these latter must be evicted more frequently from the CS.

We also observe that the FP information is static, i.e., it is not decreased by caching nodes when answering requests. Indeed, NDN Data packets are immutable [36]: if some information in the packet changes, the producer must generate a new packet and sign it. Under such conditions, cache inconsistency can still occur. As an example, we consider a scenario where an RSU monitors the average speed on a certain road and produces new Data packets named */RoadY/avgSpeed* every 60 s. An NDN vehicle A, implementing CEE+LRU+FP-Aware policy, requests a Data packet at time t=0 s and it is allowed to cache it for 60 s. At time t=50 s a vehicle B asks for the same content and receives it from A. According to the FP, the packet could be stored in the CS of node B for 60 s, but the residual lifetime of the packet is actually 10 s. If, at t=70 s, a vehicle C asks for the */RoadY/avgSpeed* Data packet and receives it from B, it will actually receive a stale information.

### 5.3. Quantifying Cache Inconsistency Effects

To quantify the cache inconsistency in an urban V-NDN environment, we performed a preliminary simulation campaign with ndnSIM [37]. We consider a first case, where nodes implement the legacy CEE+LRU scheme without applying the FP-Aware policy, and a second one where, instead, the FP-Aware policy is implemented.

The simulation scenario is a Manhattan Grid of size 1 km${}^{2}$ with 2 lanes per direction, where 100 vehicles move at speeds ranging between 20 and 40 km/h, according to the Simulation of Urban MObility (SUMO) model [38]. One RSU acting as the original producer of transient contents is deployed in the middle of the topology. Vehicles and RSU interact through the broadcast transmissions of Interest/Data packets, according to the V-NDN forwarding strategy in [21]. IEEE 802.11 is considered to be the access layer technology.

We consider a catalog of 10,000 transient contents, each one composed of 100 1kbyte-long Data packets. As with [16], we assume that nodes have the same storage space, which summed up accounts for 1% of the overall content catalog size. We also assume that 20 vehicles are selected as consumers, and the content request pattern follows a Zipf distribution [39], which is commonly used to model content popularity in the current Internet, NDN networks and VANETs [33,40,41].

Given a catalog of content items, the Zipf distribution assumes that the access probability of the $i^{th}$, 1≤i≤m, content item is represented as:

(1) $$P\left(i,\alpha ,m\right)=\frac{1/i^{\alpha }}{\sum_{z=1}^{m}\left(1/z^{\alpha }\right)}$$

where the α exponent, which is typically denoted as *skewness parameter*, characterizes the distribution popularity. The higher the value of α, the higher the number of requests concentrated on a few (popular) contents.

In this simulation, we consider a skewness parameter α equal to 1 or 2. Content requests start asynchronously: the time between two consecutive Interest transmissions for the first Data packet by different consumers is exponentially distributed with rate λ=0.3request/sec.

Two distinct metrics are reported in this preliminary evaluation stage:

- the *cache hit ratio*, computed as the ratio, in percentage, between the received Interests satisfied by the local CS and the total number of received Interests;
- the *cache inconsistency*, computed as the ratio, in percentage, between the received Data packets that were expired and the total number of received Data packets.

Table 1 reports the results averaged over 15 runs, in presence of CEE+LRU, when considering a first case where all the Data packets have the FP set to 20s, and a second case where the parameter is set to 10s. It can be observed that, reasonably, the cache hit ratio largely increases when parameter α passes from 1 to 2, since a larger number of requests are issued for the same popular contents. Not surprisingly, the lower the FP the higher the cache inconsistency, which can reach even 62.06% when α=2 and FP = 10s. This means that more than half of the cached Data packets are actually expired, but they are not removed from the CS, since the caching system is not able to recognize it, and only the LRU replacement policy applies. It is also worth noticing that the higher is α, the higher is the cache inconsistency, since the dissemination of stale cached packets is higher over the shared broadcast medium.

Table 2 shows the cache hit ratio and inconsistency metrics, when instead considering the CEE+LRU+FP-Aware policy in the same scenario. Compared to Table 1, it can be observed that reported values are considerably lower. Indeed, thanks to the FP-Aware policy, the nodes can cache contents for a time equal to their FP and, when this latter expires, the packets are removed from the CS. As a result, compared to the previous case, the cache hit ratio is lower and the cache inconsistency reduces, although a not negligible percentage, in the range 3–9%, is still present.

We can conclude that the vanilla caching system in V-NDN is not able to guarantee cache consistency in presence of transient contents. This motivates our proposal in the next Section.

### 5.4. Freshness-Driven Caching (FDC) Strategy

In this Section, we present a simple and fully distributed freshness-driven caching strategy that V-NDN nodes can apply without exchanging any additional control message. FDC is designed with two main targets in mind: to avoid cache inconsistency effects and to privilege caching contents with a longer residual lifetime.

**Figure 3:**
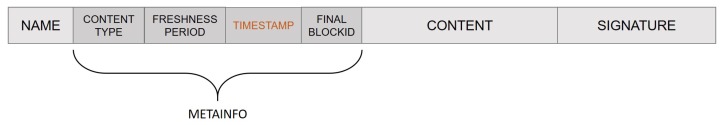
New structure of the NDN Data packet.

To overcome the cache inconsistency of the FP-Aware policy, FDC requires that information about the generation time (i.e., a timestamp) is added in the Data packet by the producer. The timestamp can be included as an additional MetaInfo field of the packet header, after the FP information, see Figure 3.

Instead of using the FP information for setting the time a content can remain in the CS, the caching system must consider the residual freshness period (RFP), defined as:

(2) RFP=FP+timestamp−currentTime

where *currentTime* is the instant the vehicle is receiving the Data packet. A proper computation of the *RFP* parameter is ensured by the fact that all vehicles maintain strict synchronization with the Coordinated Universal Time (UTC) that can be acquired from the Global Navigation Support System (GNSS) [42].

When caching the Data packet, the node sets a timeout equal to *RFP*. When the timeout expires, the content is erased from the CS and, therefore, cache inconsistency is avoided. In FDC, RFP-based eviction is integrated with a traditional replacement policy, such as LRU. Therefore, in principle, Data packets could be erased also before the *RFP* timeout expires.

FDC also implements a probabilistic caching decision strategy based on the RFP value: the target is to cache with higher probability the Data packets with a longer residual lifetime. The distinction between long- or short-lasting packets is done by setting a dynamic threshold value, $Th_{RFP}$, obtained as the exponential weighted moving average (EWMA) of the RFP values carried by the received Data packets, regardless of their senders.

More specifically, when a Data packet, *i*, traverses a node, it is cached with probability $P_{c}\left(i\right)$, which is computed as:

(3) $$P_{c}\left(i\right)=\left\{\begin{array}{cc} 1 & if\;RFP_{i}\geq Th_{RFP_{i}}\\ \frac{RFP_{i}}{Th_{RFP_{i}}} & \;otherwise \end{array}\right.$$

where:

- $Th_{RFP_{i}}$ is the current value of the threshold, as available at the reception of packet *i*;
- $RFP_{i}$ is the RFP value computed starting from the fields carried by packet *i*.

After the caching decision is taken, regardless of its outcome, the node updates the threshold as follows:

(4) $$Th_{RFP_{i+1}}=\left(1-\beta \right)Th_{RFP_{i}}+\beta \cdot RFP_{i}$$

where parameter β∈(0,1) is set to 0.125 to avoid large fluctuations in the estimation and give more relevance to the historical values in front of the instantaneous ones, as commonly agreed in multiple works in the literature, e.g., [43,44].

At the reception of a subsequent Data packet, i+1, the novel value $Th_{RFP_{i+1}}$ will be used for the caching decision.

## 6. Performance Evaluation

To assess the performance of FDC, we performed a simulation campaign in two distinct vehicular scenarios: the same urban topology described previously, and a highway topology, which consists of a 2 km-long highway road segment, where 100 vehicles move at a maximum speed of 90 km/h. In both scenarios, we assume that a RSU in the middle of the topology acts as original producers of transient contents. The *FreshnessPeriod* of Data packets varies uniformly in the range [5–100] s to match a realistic and heterogeneous data traffic pattern.

CEE and Random Caching (RC) with probability p=0.5 are considered to be benchmark schemes. They were selected as the most representative baseline solutions in the literature for V-NDN. As with FDC, they have the virtue of simplicity and incur no overhead, being completely autonomous. This is a crucial feature in the vehicular domain. For the sake of a fair comparison, all the schemes implement LRU coupled with RFP-based replacement. By doing so, cache inconsistency is always null. The proposal as well as the benchmark schemes were implemented in ndnSIM [37].

The main simulation settings are reported in Table 3.

In addition to the cache hit ratio, the following metrics are considered:

- *Content retrieval delay*, computed as the average time for retrieving a content.
- *Number of hops*, computed as the average number of hops travelled by the Interest packets for retrieving the content.
- *Number of Data packets* as the total number of Data packets broadcasted by vehicles in the simulation. It, therefore, includes also re-transmitted and redundant packets.

Results are averaged over ten independent runs and reported with 95% confidence intervals.

### 6.1. Urban Scenario

**Figure 4:**
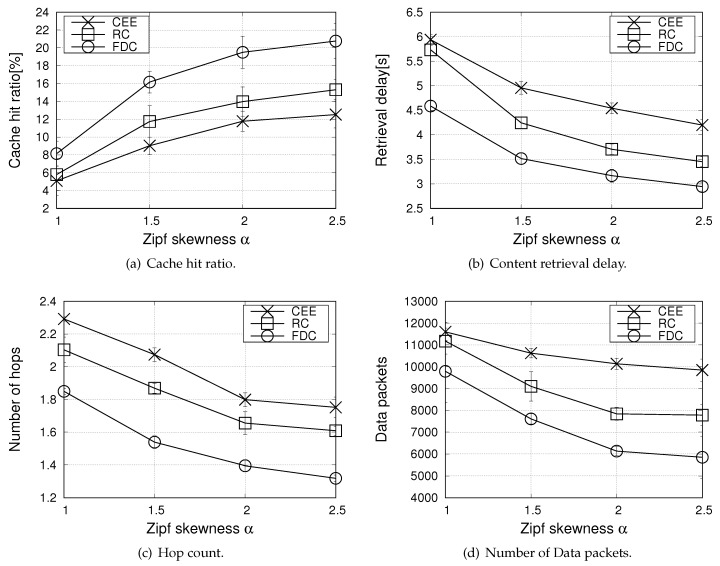
Performance metrics in the urban scenario, when varying the Zipf skewness parameter α (number of consumers equal to 20).

The first set of results in Figure 4 focuses on the urban scenario, when varying the Zipf skewness parameter, α, in the range 1, 1.5, 2, 2.5 to model different content popularity distributions.

Figure 4a reports the cache hit ratio (the metric equally applies for the different lifetimes of contents). Not surprisingly, the CEE strategy exhibits the poorest performance. Due to the broadcast nature of the wireless medium, in fact, many neighbouring vehicles are likely to receive the same Data packets at the same time instant and they all cache the same information, with a neat penalty in terms of content diversity. Better performance is obtained with the RC strategy which, by introducing the probabilistic caching decision, increases the content diversity in the network and facilitates cache hits.

In FDC, a probabilistic decision is also foreseen, but it prioritizes caching of contents with a longer residual lifetime, hence it increases the cache hit ratio, and, consequently, reduces the content retrieval delay, as shown in Figure 4b. The cache hit ratio metric increases for all the compared schemes when the Zipf skewness parameter increases. Indeed, as α increases, requests from multiple consumers concentrate on a few contents from the catalog and the more popular contents are kept in the cache. This increases the chance for a request to be satisfied and a lower number of hops for the content retrieval is experienced, see Figure 4c.

Figure 4d shows that, as a further benefit, the proposal allows reducing the number of Data packets which are exchanged into the network compared to the benchmark schemes. The largest load is experienced by the CEE scheme and it can be observed that the latter one is the less sensitive strategy to the Zipf skewness parameter. This happens because, by caching all incoming contents indiscriminately, CEE unavoidably results in a high redundancy. When multiple nearby vehicles receive a broadcast content request and have a match in the CS, they all try to answer with the Data packet. Although V-NDN implements collision avoidance techniques, based on defer times and overhearing, hidden terminal phenomena cannot be completely avoided and multiple redundant packet are transmitted.

**Figure 5:**
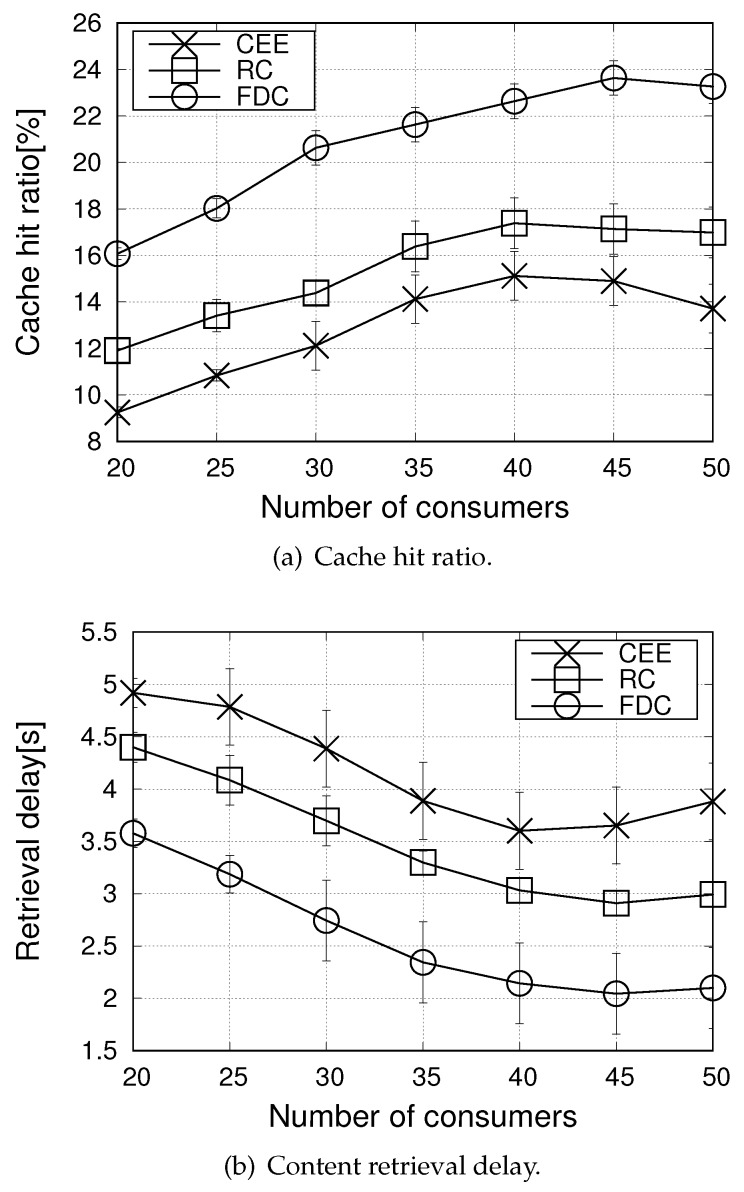
Performance metrics in the urban scenario, when varying the number of consumers (α=1.5).

Figure 5 reports the cache hit ratio and retrieval delay metrics when varying the number of consumers from 20 to 50, for the Zipf skewness parameter α set equal to 1.5. It can be observed that performance improves as the number of consumers increases from 20 to 40. In this case, the value of α ensures that many requests by multiple consumers concentrate on the more popular contents, thus, ensuring a high cache hit ratio and a low retrieval delay. Moreover, content sharing is facilitated due to the broadcast nature of the wireless medium. The supremacy of the proposed solution compared to the benchmark schemes is confirmed also under such settings.

Notwithstanding, no more improvements are experienced when the number of consumers reaches the value of 40. Indeed, with a higher number of consumers also the number of distinct contents increases: more contents are requested that are less popular, which implies a higher traffic congestion in the network and a lower cache hit ratio.

### 6.2. Highway Scenario

**Figure 6:**
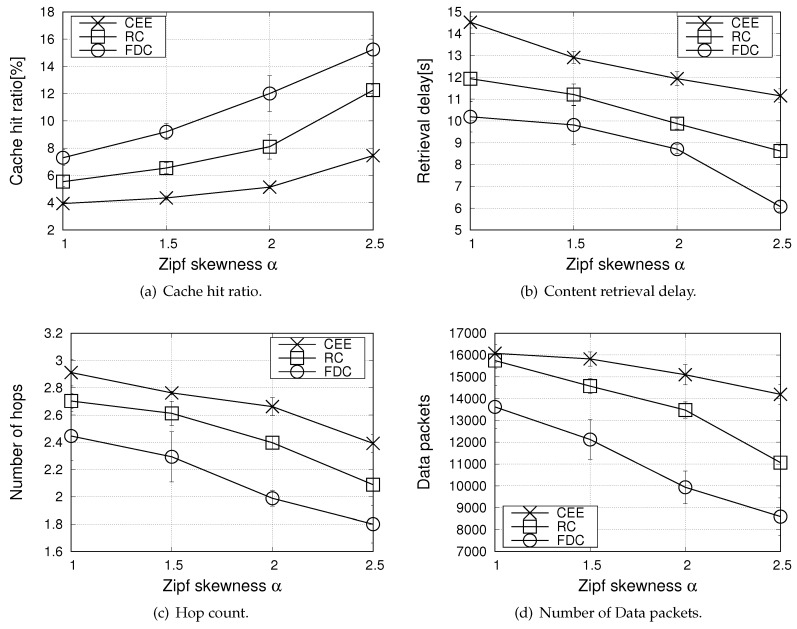
Performance metrics in the highway scenario, when varying the Zipf skewness parameter α (number of consumers equal to 20).

The second set of results, in Figure 6, focuses on the highway scenario, when varying the Zipf skewness parameter α in the range 1, 1.5, 2, 2.5.

The same trends can be observed already seen for the urban scenario. First, the proposed solution, FDC, outperforms the benchmark schemes, under all settings. Second, performance gets better for all schemes as the α parameter increases. The main difference regarding the previous scenario is that all solutions exhibit slightly worse performance. For instance, in Figure 6b, it can be seen that the lowest retrieval delay is 6.1s for FDC when α=2.5, while it was about 3s in the urban scenario in the same settings. This has to be ascribed to the topology and the higher vehicle speed which make contact times among vehicles shorter, hence also reducing the caching events. As a result, the number of hops increases as well as the delay in retrieving contents which entails the exchange of more Data packets.

**Figure 7:**
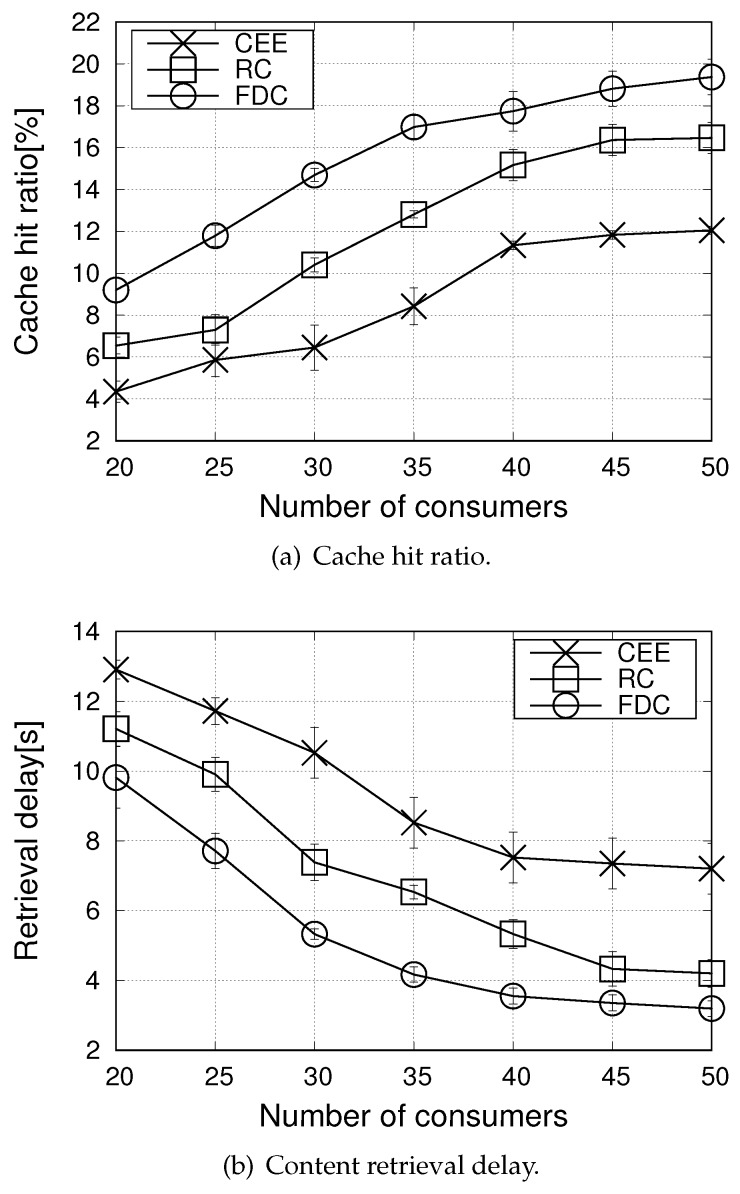
Performance metrics in the highway scenario, when varying the number of consumers (α=1.5).

As shown in Figure 7, the increasing number of consumers affects the achieved performance, similarly to the urban scenario. However, the trend of the experienced improvements in terms of both cache hit ratio and retrieval delay gets steeper as the number of consumers increases, compared to the urban scenario. Indeed, less congestion is experienced in this topology, due to the higher volatile nature of connectivity and the smaller size of the one-hop neighborhood per vehicle.

## 7. Conclusions

In this paper we investigated the issues related to the caching of transient contents in V-NDN. We conceived a novel autonomous caching strategy, FDC, in which the caching decision is taken according to the content lifetime. The solution is meant to be as compliant as possible with the legacy NDN caching routines and not to add additional signaling overhead, which could uselessly overwhelm highly dynamic vehicular links. The addition of a single field, i.e., the timestamp, to the NDN Data packet is foreseen to allow nodes infer the actual residual lifetime of contents.

Simulation results conducted under realistic mobility and data pattern settings confirm the supremacy of the proposal against two representative benchmark solutions in terms of valuable metrics, i.e., content retrieval latency, cache hit ratio, number of hops, and exchanged Data packets.

FDC can be integrated in traditional caching approaches in order to let them deal with transient content. As future work, we plan to investigate this aspect and to design a more sophisticated caching strategy, e.g., relying on content popularity and topological information of cachers, besides content freshness.

## Figures and Tables

**Table 1:** Cache hit ratio and inconsistency metrics in presence of CEE+LRU policy, when varying the Zipf skewness α and the FP parameter.

	Hit Ratio [%]	Inconsistency (FP = 20s)	Inconsistency (FP = 10s)
α=1	16.5%	19.42%	25.96%
α=2	38.2%	50.65%	62.06 %

**Table 2:** Cache hit ratio and inconsistency metrics in presence of CEE+LRU+FP-Aware policy, when varying the Zipf skewness parameter α and the freshness period.

	Hit Ratio [%]	Inconsistency (FP = 20s)	Inconsistency (FP = 10s)
α=1	9.12%	3.81%	5.46%
α=2	20.27%	7.01%	9.52 %

**Table 3:** Main simulation settings.

Parameter	Value
Content catalog size	10,000 contents
Content size	100 Data packets
Data packet size	1000 bytes
Content Popularity	Zipf distributed with α∈[1–2.5]
Propagation	Nakagami fading
Scenario	Urban topology (Manhattan Grid of size 1 km${}^{2}$)
	Highway topology (2 km-long highway road segment)
Number of vehicles	100
Number of consumers	20–50
Number of producers	1 RSU
